# Importance Calculation of Customer Requirements for Incremental Product Innovation

**DOI:** 10.3389/fpsyg.2021.633472

**Published:** 2021-06-11

**Authors:** Lisha Geng, Xiaofei Shi, Liran Zu, Meiqun Chai, Jinge Xing

**Affiliations:** ^1^School of Business Administration, Hebei University of Economics and Business, Shijiazhuang, China; ^2^Zhengding Advanced Normal College of Hebei, Shijiazhuang, China

**Keywords:** incremental product innovation, importance, customer requirements, interval grey number, multi-dimensional vector cosine method

## Abstract

Incremental product innovation is achieved by finding and solving problems of existing products. The importance of customer requirements reflects the severity of existing product problems, which points out the direction for incremental product innovation. In this research, the calculation process of customer requirement importance mainly includes three steps. Firstly, from the perspective of customers, the improvement gap analytical method is used to obtain the improvement and original importance of customer requirements by measuring customer perceived satisfaction and dissatisfaction. Secondly, from the perspective of industry experts, an improved interval grey number ranking method is proposed to calculate the basic importance of customer requirements, which can deal with the inadequate problem of the data provided by experts due to the limited number of experts. Finally, a multi-dimensional vector cosine method, which avoids the interference of subjectivity of importance weight calculation to the final importance, is proposed to integrate the importance data provided by customers and experts. A case of a water purifier is considered to illustrate the validity of the proposed process. This research improves existing calculation methods and proposes an integrated calculation process from three dimensions to calculate the final importance of customer requirements effectively.

## Introduction

A gap is formed between existing products and customers’ ideal products because of the constant changes of customer requirements and products’ flaws that continuously appear in the usage process of products (Nuscheler et al., 2019). The feelings of customers with regard to existing products varies from satisfaction to dissatisfaction, which has led to the generation of customer requirements for improving existing products. The objective of incremental product innovation is to constantly shorten the gap between existing products and customers’ ideal products and meet the requirements of current and potential customers (Lin et al., 2013).

Incremental product innovation allows enterprises to innovate by solving the most concerning problems reported by customers using limited resources (Arnett et al., 2018). The purpose of incremental product innovation is to improve customer satisfaction by innovating existing products with reasonable allocation of resources. The customer requirement-driven process is a key process in the early stages of incremental product innovation (Mourtzis et al., 2018). The innovative scheme produced to this stage directly affects the detailed design of the product and its commercial implementation.

Previous studies calculate the importance of customer requirements from a single perspective; this situation results in the deviation from the real needs of customers and eventually leads to the failure of product innovation. In September 2003, a company in China innovated the first washing powder-free ionised washing machine (Kong and Han, 2016). The washing machine was innovated according to the customers’ requirements for washing clothes without using washing powder. However, this washing machine was eventually withdrawn from the market because of a cold market reaction. The main reason for the failure of the innovated washing machine was the lack of accurate calculation of the importance of customer requirements for washing machines. Although washing clothes without washing powder was a requirement for customers, it was not more important than other requirements; thus, meeting this requirement would not improve customer satisfaction with the washing machines. Given this situation, the importance of customer requirements must be accurately calculated to ensure the effect of innovated products. Thus, we attempt to address the following research question:

RQ: How can we accurately calculate the importance of customer requirements to ensure that the most serious problems existing in current products can be identified, and incremental product innovation can be performed in the right direction on the basis of customer requirement importance?

The remainder of this paper is organised as follows. Section 2 provides a full review of the calculation of the importance of customer requirements. Section 3 proposes the integrated calculation method for evaluating the importance of customer requirements. Section 4 presents a case study of a water purifier in China. Section 5 provides a discussion. Finally, Section 6 concludes the paper.

## Literature Review

### Importance Calculation of Customer Requirements for Incremental Product Innovation

Customer requirements provide the information source for product innovation. In the early stage of innovation, the direction of product innovation is obtained through customer requirements. The driving force of customer requirements has a greater impact on the incremental product innovation (Wang et al., 2016). Manufacturers make partial improvement on products on the basis of existing knowledge and ability to meet the requirements of customers; therefore, customers are regarded as the source of incremental product innovation. Successful incremental innovation comes from the precise positioning of customers’ requirements. Integrating the importance of customer requirements into incremental product innovation process can achieve good results, especially in the creative generation and conceptual design stage of the product. This mechanism can effectively avoid the phenomenon of blind innovation due to ignoring customer requirements. Enterprises need to calculate the importance of customer requirements as accurately as possible and consider the factors affecting the importance of customer requirements to ensure that the effect of incremental product innovation can more fully meet the preferences of customer requirements (Wang et al., 2018).

A close relationship exists between the importance of customers and their satisfaction (Madzík et al., 2019). Existing literature uses the Kano model to link customer satisfaction with the calculation of customer requirement importance (Gupta and Shri, 2018). However, such work does not consider the gap of customer satisfaction between existing products and ideal products. Accordingly, the improvement of customer satisfaction by meeting a certain customer demand cannot be predicted. If a certain customer requirement is important, but the existing products have already fully met this requirement and have high customer satisfaction, then the improvement of the requirement is low, which is a new concept proposed in this research. Hence, meeting this customer requirement will not be the focus of incremental product innovation. The customer requirement with high improvement but low importance is also not the object of incremental product innovation. For example, if the mobile phone cannot meet the customer’s call requirement, then customers must be very dissatisfied; therefore, customers’ call requirement for mobile phone is very important. However, when customers think that no gap exists between the call function of existing mobile phone and the ideal phone, customers are already very satisfied with the call function of existing phone. Furthermore, meeting the call requirement of mobile phone cannot significantly improve customer satisfaction. Thus, meeting customer call requirement for phone will not be the key concern of the incremental innovation of phone.

On this basis, the improvement for customer satisfaction by meeting a customer requirement should be considered an important index for calculating the final importance of a customer requirement for incremental product innovation. However, the above point has not been studied in the existing literature. Hence, the improvement of customer requirements is taken in this research as an important index for calculating the importance of customer requirement by measuring how much customer satisfaction is improved by meeting a customer requirement.

### Data Sources for Obtaining the Importance of Customer Requirements

The two types of data sources for obtaining the importance of customer requirements directly are as follows: the first type is provided by customers (Li et al., 2018; Zheng et al., 2019), and the other one is disclosed by experts in a certain industry (Kong and Hao, 2001; Xie et al., 2016).

However, neither data source is completely reliable. Customers often confuse the importance of customer requirements with the need to improve them; therefore, data gained from customers should be further refined. Customers often do not know what they really want; therefore, complete obedience to the importance data of customer requirement provided by customers easily results in the deviation from the developing prospects of the industry, which is not conducive to the long-term development of enterprises.

Experts include product designers, lead users of the product, product maintenance personnel and industry experts who can indicate the importance of customer requirements from the point of view of product design (Xie et al., 2016).

For example, the incremental innovation of mobile phones from functional phones to smart phones is inseparable from the contribution of industry experts who can predict the real requirements of customers through the aspect of product development. On this basis, we also need to consider the analysis of customer requirements by industry experts to effectively realise incremental product innovation. Accordingly, the data sources provided by customers and experts may be combined to calculate the importance of customer requirements in a more comprehensive and systematic manner (Kong and Hao, 2001; Xiong, 2012). However, the current research work subjectively assigns the weight of data provided by experts and customers (Li et al., 2018), which cannot avoid the impact of human subjective assignment on real data. Therefore, this study proposes multi-dimensional vector cosine method to objectively assign the weight of different importance data.

### Uncertainties in the Calculation of the Importance of Customer Requirement

The importance of customer requirements is calculated as an artificial input, which is inevitably subjective and uncertain. Fuzzy theory has been employed to deal with this uncertainty (Masud and Dean, 1993; Che and Lin, 1998). However, the membership function needs to be artificially determined in advance in fuzzy theory. Therefore, the uncertainties cannot be completely dealt with.

The rough set theory has also been used to solve the uncertainties. Although the rough set theory can overcome the shortcomings of fuzzy theory, decision makers need to calculate the upper and lower approximation limits and the boundary regions of rough numbers; thus, the calculation process is complicated. In addition to the lack of relevant data processing software, rough set theory is not suitable for calculating importance.

The grey system theory has been applied to solve the uncertainties. Deng Julong founded the grey system theory in 1982; this theory can deal with the uncertainty problems caused by inadequate data (Deng, 1983). Therefore, the interval grey numbers are used to solve the problem (Liu and Cheng, 2016). However, the interval grey numbers need to be ranked to calculate the importance of customer requirements with interval grey numbers.

The methods for ranking interval grey numbers can be found in the existing literature; these methods include the method based on set theory (Liu and Dang, 2010), possibility function (Wang, 2012) and ideal interval grey number. A common problem of the three methods is that only two interval grey numbers are compared at a single time. When the number of interval grey numbers representing customer requirements is large, the calculation process is complicated and time consuming. Thus, a new method for simultaneously ranking multiple interval grey numbers is proposed in this research to solve this problem.

In summary, the existing research on the importance calculation of customer requirements still has the following three shortcomings: (1) When using customer satisfaction to measure the importance of customer requirements, the measurement of customer satisfaction between the current product and the ideal product is ignored; thus, such mechanism is unable to measure the impact of meeting a certain customer requirement on the customer satisfaction of the product. Accordingly, the customer satisfaction of incremental product innovation cannot be effectively improved. (2) In realising incremental product innovation, the data from the perspectives of customers and industry experts must be integrated, and how to objectively and effectively integrate the data from the two perspectives is a problem to be solved; (3) How to effectively deal with the uncertainty caused by inadequate data due to the small number of customers in the process of customer requirement assignment is a problem to be solved.

To solve the above problems, this study firstly proposed the concept of improvement of customer requirement, which is used to measure the improvement of customer satisfaction by meeting a certain requirement. The improvement is taken as one of the indicators in the calculation of customer requirement importance to ensure that product incremental innovation can improve customer satisfaction to a greater extent. Secondly, the multi-dimensional vector cosine method is proposed to realise the effective integration of multi-dimension of importance for calculating the final importance of customer requirement, which can objectively assign the weight of each dimension of importance by using mathematical methods. Thirdly, an improved interval grey number ranking method is proposed to solve the uncertainty of inadequate data problem of low number of experts, which simultaneously ranks multiple interval grey numbers. Thus, the ranking of interval grey numbers is more convenient and accurate.

## Research Framework

**FIGURE 1:**
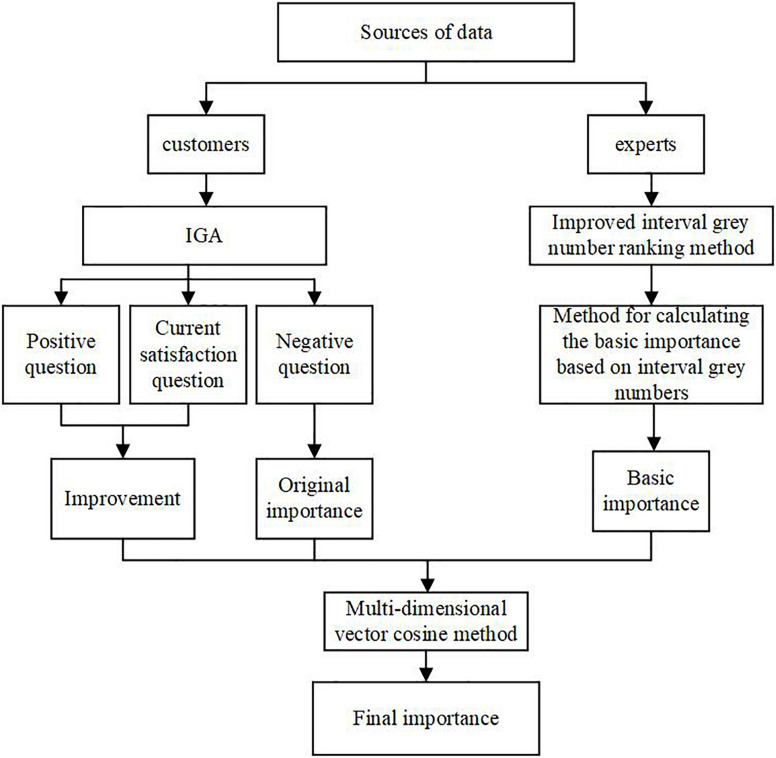
Integrating calculation of the final importance of customer requirement.

The calculation of the importance of customer requirements for incremental product innovation is an integrated calculation process. An overview of the proposed methodology is shown in Figure 1.

From the perspective of customers, the improvement gap analysis (IGA) is used to measure the original importance and the improvement of customer requirements on the basis of three questions: positive, current satisfaction and negative questions.

From the perspective of experts, an improved interval grey number ranking method is proposed to calculate the basic importance of customer requirements, which can deal with the inadequate data provided by the limited number of experts.

Finally, a multi-dimensional vector cosine method is employed to integrate the importance of customer requirement provided by customers and experts. This method objectively combines the original importance, improvement, and basic importance of customer requirements.

### IGA

In the Kano model, two contexts are set to measure customer satisfaction: one where the product possesses certain attributes or has high performance in certain aspects (positive question) and the other one where the product does not possess certain attributes or has low performance in certain aspects (negative question) (Kano et al., 1984). However, the Kano model cannot evaluate customers’ satisfaction with the current product. Therefore, the gap in customer satisfaction between the current product and the customers’ ideal product cannot be measured.

IGA was suggested by Tontini on the basis of the Kano model to measure customer satisfaction (Tontini et al., 2014), which used positive, negative, and current satisfaction questions to calculate customer satisfaction or dissatisfaction in three situations. The question template is summarised in Table 1, wherein 1 to 5 represents the corresponding score of customer perceived satisfaction or dissatisfaction.

**TABLE 1:** Question template of IGA.

Category	Question	VD	D	N	S	VS
Positive question	When the maintenance cost of water purifier is low, how do you feel?	1	2	3	4	5
Negative question	When the maintenance cost of water purifier is high, how do you feel?	5	4	3	2	1
Current satisfaction question	How do you feel about the maintenance cost of your water purifier?	1	2	3	4	5

*VD = very dissatisfied; D = dissatisfied; N = neutral; S = satisfied; VS = very satisfied.*

In this research, IGA is used to obtain the improvement and the original importance of customer requirements provided by customers through customers’ responses to three questions. The gap between the satisfaction perceived from the ideal product (positive question) and the current product (current satisfaction question) is defined as the improvement of customer requirement. The negative question is used to measure the expected dissatisfaction of customers when the product does not meet a customer requirement. When a customer requirement is not met, the more unsatisfied customers feel, the more important this requirement is. The original importance is determined by the customer’s perceived dissatisfaction.

This study assumes that some customers participate in the survey of the importance of customer requirements, where *n* and *m* represent the number of customers and customer requirements, respectively. With regard to the positive question, the satisfaction perceived by the *j*_*th*_ customer by meeting the *i*_*th*_ customer requirement is represented by *Pj i*. The corresponding customer satisfaction perceived with the *i*_*th*_ customer requirement under the positive ideal state of the product, expressed by *AP*_*i*_, is calculated with Eq. (1). In terms of the current satisfaction question, the satisfaction perceived by the *j*_*th*_ customer by meeting the *i*_*th*_ customer requirement is expressed by *CS^*j*^_*i*_*. The corresponding customer satisfaction, expressed by *ACS*_*i*_, is calculated by Eq. (2). *IG*_*i*_ and *W*_*IGi*_ that represent the improvement of customer requirement and its weight are calculated by Eqs. (3) and (4), respectively. As regards the negative question, the dissatisfaction perceived by the *j*_*th*_ customer without meeting the *i*th customer requirement is represented by *N^*j*^_*i*_*. The corresponding customer dissatisfaction with the *i*_*th*_ customer requirement under the negative ideal state of the product is expressed by *AN*_*i*_. *AN*_*i*_ and *W_*A*__*N*__*i*_* that represent the original importance and its weight are calculated by Eqs. (5) and (6), respectively.

(1) $$AP_{i}=\frac{1}{n}\sum_{j=1}^{n}P_{i}^{j},\;i=1,2,\cdots ,m.$$

(2) $$ACS_{i}=\frac{1}{n}\sum_{j=1}^{n}CS_{i}^{j},\;i=1,2,\cdots ,m.$$

(3) $$IG_{i}=AP_{i}-ACS_{i},\;i=1,2,\cdots ,m.$$

(4) $$W_{IG_{i}}=\frac{IG_{i}}{\sum_{i=1}^{m}IG_{i}},\;i=1,2,\cdots ,m.$$

(5) $$AN_{i}=\frac{1}{n}\sum_{j=1}^{n}N_{i}^{j},\;i=1,2,\cdots ,m.$$

(6) $$W_{AN_{i}}=\frac{AN_{i}}{\sum_{i=1}^{m}AN_{i}},\;i=1,2,\cdots ,m.$$

### Grey Theory Preliminaries

#### Improved Interval grey Number Ranking Method

The basic importance of customer requirement is calculated with the data provided by experts, who tend to comprehensively measure the importance. In view of the adequate data provided by limited experts, interval grey numbers are employed by experts to evaluate the basic importance of customer requirements (Liu and Cheng, 2016).

The definitions of grey numbers are as follows:

**Definition** 1. Assuming that *x* is an element in a complete set denoted as *X*, the grey set *G* on *X* is determined by the upper membership function $\bar{\mu }$_*G*_(*x*) and the lower membership function $\underline{\mu }$_*G*_(*x*).

(7) $$\left\{ \begin{array}{c} {\bar{\mu }}_{G}\left(x\right):x\to \left[0,1\right]\\ {\underline{\mu }}_{G}\left(x\right):x\to \left[0,1\right] \end{array}\right.,\begin{array}{cc} & x\in X \end{array},$$

**Definition** 2. Grey number is a grey set with uncertain information, which is defined as ⊗*G*. If the lower bound of ⊗*G* is known, then ⊗*G* is defined as the lower bound grey number; if the upper bound of ⊗*G* is known, then ⊗*G* is called the upper bound grey number.

(8) $$\begin{array}{c} ⊗G=\left(-\infty ,\bar{G}\right]\\ ⊗G=\left[\underline{G},\infty \right) \end{array}$$

**Definition** 3. If the upper and lower bounds of ⊗*G* are known, then ⊗*G* is called the interval grey number.

(9) $$⊗G=\left[\underline{G},\bar{G}\right]$$

**Definition** 4. Assume that two interval grey numbers ⊗*G*_1_ and⊗*G*_2_ are present. The addition, subtraction, multiplication, division, and multiplication algorithms of these numbers are shown as Eqs. (10)–(14), respectively.

(10) $$\begin{array}{c} ⊗G_{1}=\left[\underline{G_{1}},\bar{G_{1}}\right]⊗G_{2}=\left[\underline{G_{2}},\bar{G_{2}}\right]\\ ⊗G_{1}+⊗G_{2}=\left[\underline{G_{1}}+\underline{G_{2}},\bar{G_{1}}+\bar{G_{2}}\right] \end{array}$$

(11) $$⊗G_{1}-⊗G_{2}=\left[\underline{G_{1}}-\bar{G_{2}},\bar{G_{1}}-\underline{G_{2}}\right]$$

(12) $$\begin{array}{c} ⊗G_{1}\cdot ⊗G_{2}=[\min\left(\underline{G_{1}G_{2}},\underline{G_{1}}\bar{G_{2}},\bar{G_{1}}\underline{G_{2}},\bar{G_{1}G_{2}}\right),\\ \;\max\left(\underline{G_{1}G_{2}},\underline{G_{1}}\bar{G_{2}},\bar{G_{1}}\underline{G_{2}},\bar{G_{1}G_{2}}\right)] \end{array}$$

(13) $$\begin{array}{c} \frac{⊗G_{1}}{⊗G_{2}}=\left[\underline{G_{1}},\bar{G_{1}}\right]\cdot \left[\frac{1}{\bar{G_{2}}},\frac{1}{\underline{G_{2}}}\right]\\ =\left[\min\left(\frac{\underline{G_{1}}}{\bar{G_{2}}},\frac{\underline{G_{1}}}{\underline{G_{2}}},\frac{\bar{G_{1}}}{\bar{G_{2}}},\frac{\bar{G_{1}}}{\underline{G_{2}}}\right),\max\left(\frac{\underline{G_{1}}}{\bar{G_{2}}},\frac{\underline{G_{1}}}{\underline{G_{2}}},\frac{\bar{G_{1}}}{\bar{G_{2}}},\frac{\bar{G_{1}}}{\underline{G_{2}}}\right)\right] \end{array}$$

(14) $$\begin{array}{c} \left(1\right)k>0,k\cdot ⊗G_{1}=\left[k\underline{G_{1}},k\bar{G_{1}}\right]\\ \left(2\right)k<0,k\cdot ⊗G_{1}=\left[k\bar{G_{1}},k\underline{G_{1}}\right] \end{array}$$

Experts are asked to evaluate the basic importance of customer requirements with interval grey numbers; 0 represents the lowest importance, and 1 indicates the highest importance. Any interval grey number whose upper and lower bounds are composed of two numbers between 0 and 1 is selected by experts to represent the customer requirement’s basic importance. Accordingly, a group of interval grey numbers are obtained. This study supposes that the number of the interval grey numbers is represented by *m*, and the number of experts is represented by *n*. The basic importance of the customer requirements provided by experts is calculated using Eq. (15).

(15) $$CR_{i}=\frac{1}{n}\sum_{n=1}^{n}\left[\underline{G_{i}^{r}},\bar{G_{i}^{r}}\right],0<i\leq m,0<r\leq n.$$

Given that the basic importance is represented by interval grey numbers, they must be ranked, and their weights must be calculated.

**FIGURE 2:**
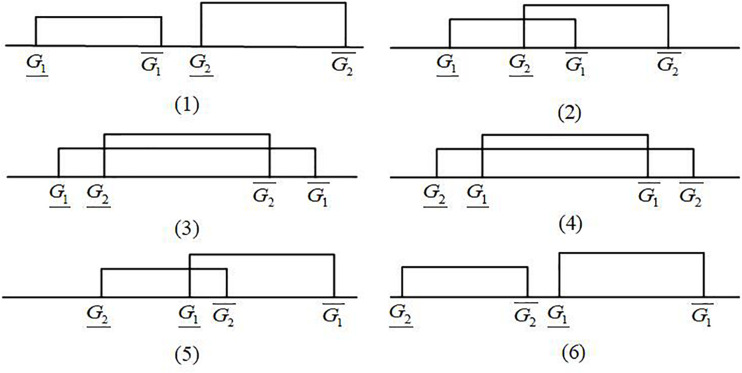
Six possible relationships between two interval grey numbers.

Six possible relationships may exist between two interval grey numbers, which are represented by ⊗*G*_1_ and ⊗*G*_2_, and they can be divided into three categories: non-intersecting, partially intersecting, and completely intersecting (Figure 2). Liu and Cheng (2016) proposed an interval grey number ranking method based on the ideal interval grey number. However, this method can only compare two interval grey numbers at a time, which requires a large number of computational tasks to rank multiple interval grey numbers. For example, if we rank eight interval grey numbers, then we need to compare 28 times.

**FIGURE 3:**
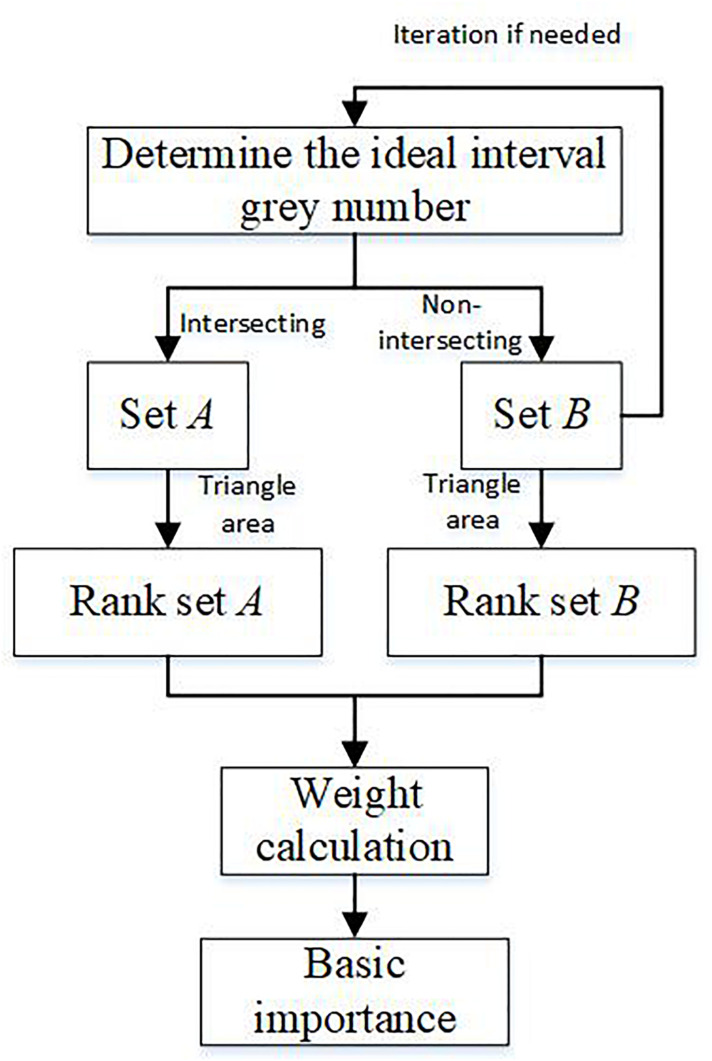
Flow chart for calculating basic importance.

An improved interval grey number ranking method is proposed in this research to effectively reduce the comparison times for ranking interval grey numbers. The flow chart for this method is shown in Figure 3, which includes the following steps.

This study supposes the existence of some interval grey numbers: ⊗*G*_1_, ⊗*G*_1_, …, ⊗*G*_*m*_. These numbers are ranked as follows:

Step 1: Determine the ideal interval grey number ⊗*G*^∗^ 1 using Eq. (16).

(16) $$⊗G_{1}^{*}=\left[\max\left(\underline{G_{1}},\underline{G_{2}},\cdots ,\underline{G_{m}}\right),\max\left(\bar{G_{1}},\bar{G_{2}},\cdots ,\bar{G_{m}}\right)\right]$$

**FIGURE 4:**
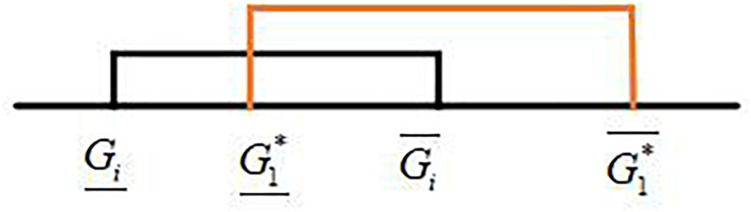
Relationship between ⊗*G*_*i*_ and the ideal interval grey number.

**FIGURE 5:**
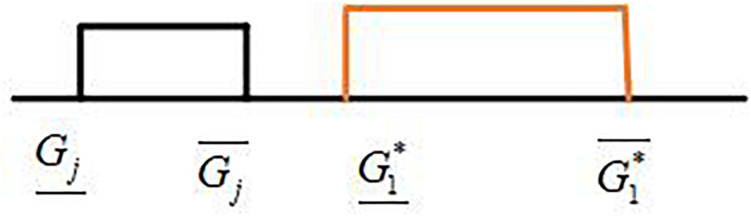
Relationship between ⊗*G*_*j*_ and the ideal interval grey number.

Compare all interval grey numbers with the ideal interval grey numbers. Then, all interval grey numbers are divided into two sets: *A* and *B* according to their relationship with the ideal interval grey number. The interval grey numbers in sets *A* and *B* are represented as ⊗*G_*i*_* and ⊗*G_*j*_*, respectively. The relationships of ⊗*G_*i*_* and ⊗*G_*j*_* with the ideal interval grey numbers shown in Figures 4, 5 are expressed by Eqs. (17) and (18), respectively.

(17) $$A=\left\{⊗G_{i}|\bar{G_{i}}>\underline{G_{1}^{*}},1\leq i\leq m\right\}$$

(18) $$B=\left\{⊗G_{j}|\bar{G_{j}}\leq \underline{G_{1}^{*}},1\leq j\leq m\right\}$$

⊗*G_*i*_* intersects with the ideal interval grey number, and ⊗*G_*j*_* does not intersect with such a number. Accordingly, the relationships between ⊗*G_*i*_* and ⊗*G_*j*_* are ⊗*G_*i*_* > ⊗*G_*j*_*, ⊗*G_*i*_*∈*A* and ⊗*G_*j*_*∈*B.*

Step 2: Rank the interval grey number ⊗*G_*i*_* in set *A*

All interval grey numbers in set *A* are simultaneously compared with the ideal interval grey numbers on the basis of the two interval grey number ranking method proposed by Liu and Cheng (2016).Thus, ⊗*G_*i*_* is ranked according to the comparison results.

**FIGURE 6:**
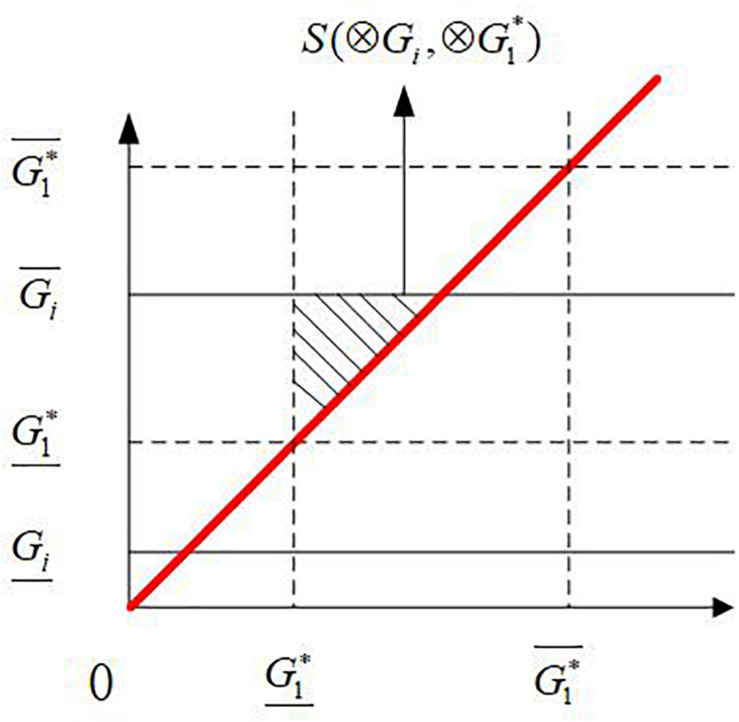
Geometric interpretation for the ranking of ⊗*G*_*i*_.

Figure 6 shows the geometric interpretation for the ranking of ⊗*G_*i*_*. Firstly, the upper and lower bounds of ⊗*G_*i*_* and ⊗*G*^∗^ 1are placed at the corresponding points on the vertical axis, which are considered the starting points to draw lines parallel to the horizontal axis. The upper and lower bounds of ⊗*G*^∗^ 1are placed at the corresponding two points on the horizontal axis, which are considered the starting point to draw lines parallel to the vertical axis. The solid lines represent the parallel lines corresponding to ⊗*G_*i*_*. The dotted lines represent the parallel lines corresponding to ⊗*G*^∗^ 1. Then, a red line is drawn; accordingly, it forms a 45°angle with the vertical and horizontal axes. Finally, an isosceles right triangle that is the shaded part representing the overlap area between ⊗*G_*i*_* and ⊗*G*^∗^ 1, is formed between the upper bound line of ⊗*G_*i*_*, the lower bound line of ⊗*G*^∗^ 1and the red line. The shaded area is the basis for ranking interval grey numbers.

The isosceles right triangle area of *S* (⊗*G*_*i*_, ⊗*G*^∗^ 1) is calculated using Eq. (19), which determines the ranking result of the interval grey numbers in set *A*.

(19) $$S\left(⊗G_{i},⊗G_{1}^{*}\right)=\frac{{\left(\bar{G_{i}}-\underline{G_{1}^{*}}\right)}^{2}}{2}$$

This study supposes that ⊗*G_*s*_* and ⊗*G_*t*_* are two interval grey numbers in set *A*; they are ranked according to the values of *S*(⊗*G*_*t*_, ⊗*G*^∗^ 1) and *S*(⊗*G*_*s*_, ⊗*G*^∗^ 1).

If *S*(⊗*G*_*s*_, ⊗*G*^∗^ 1)<*S*(⊗*G*_*t*_, ⊗*G*^∗^ 1), then ⊗*G_*s*_*<⊗*G_*t*_*.

If *S*(⊗*G*_*s*_, ⊗*G*^∗^ 1)>*S*(⊗*G*_*t*_, ⊗*G*^∗^ 1), then ⊗*G_*s*_*>⊗*G_*t*_*.

**FIGURE 7:**
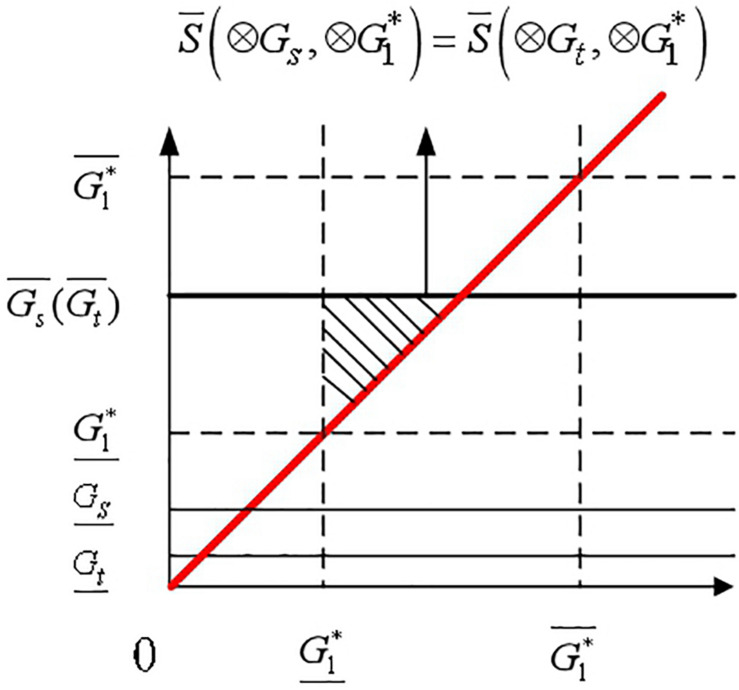
Geometric interpretation of two interval grey numbers that are equal in areas.

If multiple interval grey numbers are present in set *A*, of which the corresponding triangle areas S(⊗*G*_*i*_, ⊗*G*^∗^ 1) are equal, then the rank of these interval grey numbers is determined by the lower bounds. The larger the lower bound is, the higher it ranks. Figure 7 shows the occasion when the triangle areas formed by two interval grey numbers ⊗*G_*s*_* and ⊗*G_*t*_* are equal. ⊗*G_*s*_* and ⊗*G_*t*_* that represent the upper bounds of ⊗*G_*s*_* and ⊗*G_*t*_* are equal.

$$\bar{S}\left(⊗G_{s},⊗G_{1}^{*}\right)=\bar{S}\left(⊗G_{t},⊗G_{1}^{*}\right),⊗G_{s},⊗G_{t}\in A$$

⊗G<tG,sso⊗G<t⊗G.s

Further quantitative calculation is needed to calculate the ranks and weights of the interval grey numbers ⊗*G_*s*_* and ⊗*G_*t*_*. The triangle area formed by the grey number ⊗*G_*r*_* is represented as S(⊗*G*_*r*_,⊗*G*^∗^ 1), ⊗*G_*r*_*∈*A*. If ^–^S(⊗*G*_*s*_, ⊗*G*^∗^ 1) is less than and only less than S(⊗*G*_*r*_ ⊗*G*^∗^ 1), then the interval grey number can be distinguished by the lower bounds of ⊗*G_*s*_* and ⊗*G_*t*_*. The values of S(⊗*G*_*s*_, ⊗*G*^∗^ 1) and S(⊗*G*_*t*_, ⊗*G*^∗^ 1) were calculated using Eqs. (20) and (21); the areas corresponding to the interval grey numbers ⊗*G_*s*_* and⊗*G_*t*_* are obtained.

(20) $$\begin{array}{c} S\left(⊗G_{s},⊗G_{1}^{*}\right)=\\ \bar{S}\left(⊗G_{s},⊗G_{1}^{*}\right)+\left[S\left(⊗G_{r},⊗G_{1}^{*}\right)-\bar{S}\left(⊗G_{s},⊗G_{1}^{*}\right)\right]*\frac{\underline{G_{s}}}{\underline{G_{s}}+\underline{G_{t}}} \end{array}$$

(21) $$\begin{array}{c} S\left(⊗G_{t},⊗G_{1}^{*}\right)=\\ \bar{S}\left(⊗G_{t},⊗G_{1}^{*}\right)+\left[S\left(⊗G_{r},⊗G_{1}^{*}\right)-\bar{S}\left(⊗G_{t},⊗G_{1}^{*}\right)\right]*\frac{\underline{G_{t}}}{\underline{G_{s}}+\underline{G_{t}}} \end{array}$$

Thus, the ranking result of interval grey numbers in set *A* can be determined.

Step 3: Rank the interval grey numbers in set *B*.

The ideal interval grey number of set *B* is chosen by using Eq. (22), which is the same as step 1. Then, the interval grey numbers in set *B* are ranked according to step 2. The ranking of all interval grey numbers in sets *A* and *B* is determined from steps 1 to 3.

(22) $$⊗G_{2}^{*}=\left[\max\underline{G_{j}},\max\bar{G_{j}}\right],⊗G_{j}\in ⊗G_{B}$$

Step 4: Calculate the weights of the interval grey numbers.

The areas corresponding to the interval grey numbers in set *A* were obtained by Eq. (19). The interval grey numbers in set *B* do not intersect with the ideal interval number. The interval grey numbers in set *A* intersect with the ideal interval number; accordingly, all the interval grey numbers in set *B* are less than that in set *A*. Therefore, the minimum value in set *A* is taken as the standard to standardise all interval grey numbers in set *B* by Eq. (23).

(23) $$\begin{array}{c} \overset{\wedge }{S}\left(⊗G_{j},⊗G_{2}^{*}\right)=\min_{⊗G_{i}\in ⊗G_{A}}S\left(⊗G_{i},⊗G_{1}^{*}\right)*\frac{S\left(⊗G_{j},⊗G_{2}^{*}\right)}{\sum_{⊗G_{j}\in ⊗G_{B}}S\left(⊗G_{j},⊗G_{2}^{*}\right)}\\ ⊗G_{i}\in A,⊗G_{j}\in B \end{array}$$

With regard to ⊗*G*_1_, ⊗*G*_2_, …, ⊗*G_*m*_*, if ⊗*G_*k*_*∈*A*, 1 ≤ *k* ≤ *m*, then *S* (⊗*G*_*k*_) = *S* (⊗*G*_*k*_, ⊗*G^∗^* 1); if ⊗*G_*k*_*∈*B*, 1 ≤ *k* ≤ *m*, then *S* (⊗*G*_*k*_) = *S* (⊗*G*_*k*_, ⊗*G^∗^ 2*).

Equation (24) is applied to calculate the weights of all interval grey numbers.

(24) $$W_{k}=\frac{S\left(⊗G_{k}\right)}{\sum_{1\leq k\leq m}S\left(⊗G_{k}\right)}$$

### Multi-dimensional Vector Cosine Method

In this research, we extend the vector cosine method in 2D space (Kong and Han, 2016) into a multi-dimensional space and propose a multi-dimensional vector cosine method. The study object is regarded as a vector in a multi-dimensional space. The body diagonal, whose angles with all coordinate axes are equal, is considered as the ideal direction. The projection of the vector in the ideal direction is regarded as its integration in the multi-dimensional space.

An *n*-dimensional space with *n* lines of length one is constructed. *O* and *T*^∗^ represent the negative ideal point (the origin) and positive ideal point in the space, respectively. This study suppose that *OT*^∗^ is the ideal direction in the *n*-dimensional space, and its angles to each coordinate axis are equal.

*T* represents an arbitrary point, and *OT* is a vector in this space. θ denotes the angle of *OT* deviating from the ideal direction; ρ that denotes the projection of *OT* in the ideal direction is the integration of *OT* in the multi-dimensional space. ρ is calculated using Eqs. (25)–(28). This study supposes that *OT*_*r*_ and *OT*_*s*_ are two vectors in an *n*-dimensional space, and θ_*r*_ and θ_*s*_ represent their angles deviating from the ideal direction, respectively. *D*_*rs*_ is the distance between the projections of *OT*_*r*_ and *OT*_*s*_ in the ideal direction, which is calculated by Eq. (29).

{T=(λ1,λ2,⋯,λn)O=(0,0,⋯,0)T=*(1,1,⋯,1)

(25) $$\begin{array}{c} OT=\sqrt{{\left(\lambda_{1}-0\right)}^{2}+{\left(\lambda_{2}-0\right)}^{2}+\cdots +{\left(\lambda_{n}-0\right)}^{2}}\\ \begin{array}{cc} & =\sqrt{\lambda_{1}^{2}+\lambda_{2}^{2}+\cdots +\lambda_{n}^{2}} \end{array} \end{array}$$

(26) θ=arccos[(OT)*2+(OT)2-(TT)*2]2×OT×*OT

(27) TT=*(1-λ1)2+(1-λ2)2+⋯+(1-λn)2

(28) $$\rho^{T}=OT^{\prime }=OT\times \cos\theta$$

(29) $$D_{rs}=\left|OT_{r}\times \cos\theta_{r}-OT_{s}\times \cos\theta_{s}\right|$$

In an *n*-dimensional space, *T*^∗^ is the ideal point, and origin *O* is the negative ideal point. *D*_(T*,*O*)_, which represents the distance between the ideal point and the negative ideal point, is calculated by Eqs. (30)–(32).

(30) $$O=\left(0,0,\cdots ,0\right),\begin{array}{cc} & \theta =0 \end{array},\begin{array}{cc} & \rho^{o}= \end{array}0$$

(31) $$T^{*}=\left(1,1,\cdots ,1\right),\begin{array}{cc} & \theta =0,\begin{array}{cc} & \rho^{T^{*}}= \end{array} \end{array}\sqrt{n}$$

(32) $$D_{\left(T^{*},O\right)}=\sqrt{n}$$

**FIGURE 8:**
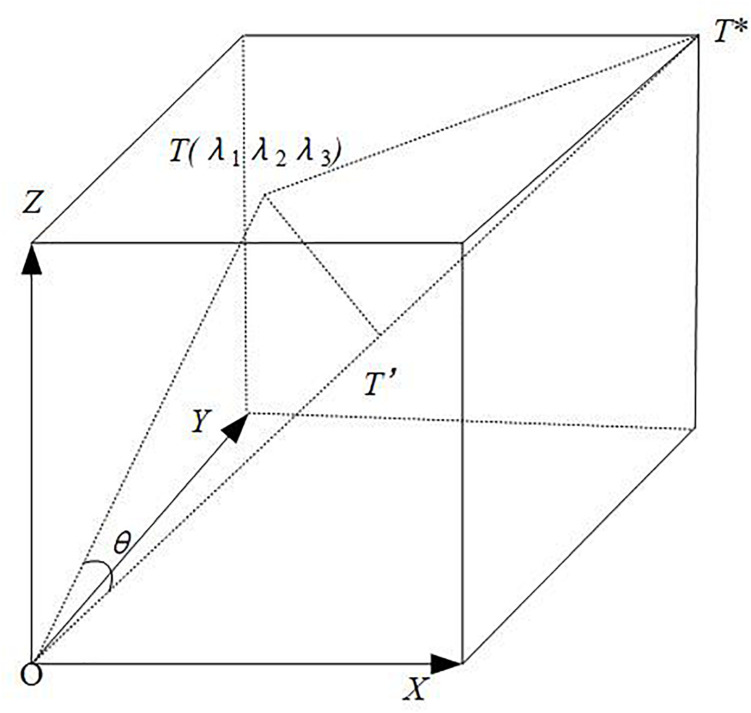
Calculation of final importance based on multi-dimensional vector cosine method.

The *X*, *Y*, and *Z* axes represent the improvement, original importance, and basic importance of customer requirement, respectively. The lengths of the axes are one; therefore, a 3D space of unit 1 is formed, as shown in Figure 8 (Geng et al., 2019). *T* denotes an arbitrary point in space. The coordinates are λ_1_, λ_2_, and λ_3_, which are all numbers between zero and one. *OT* denotes a customer requirement vector, *OT*^∗^ is the ideal direction, and θ represents the angle of customer requirement *OT* deviating from the ideal direction. *O* and *T*^∗^ represent the negative ideal and ideal points of customer requirement, respectively.

The projection of the customer requirement vector O*T* in the ideal direction is calculated by Eqs. (25)–(28). The calculation result that considers the length and angle of the customer requirement vector is the final importance of customer requirement.

The multi-dimensional vector cosine method improves the traditional method for measuring the study object solely from its numerical value. This method considers the study object as a vector; therefore, its numerical value and deviation from the ideal direction are considered. The multi-dimensional vector cosine method regards the final importance of customer demand as the ideal direction in the multi-dimensional space. Each coordinate axis in the multi-dimensional space represents a dimension of customer requirement. In this study, the original importance, improvement and basic importance of customer requirement constitute three coordinate axes in a 3D space. With the improvement of the customer requirement, the original importance and basic importance calculated above are all standardised values between zero and one. A 3D space is formed by three lines with a length of one. The projection of any vector in the ideal direction in the space that is the integration of 3D importance is regarded as the final importance of customer requirement. The weights of the three dimensions of importance, which determine the angle the customer requirement vector deviating from the ideal direction, need to be considered to calculate the integrated final customer requirement. The traditional weighted average method is disturbed by subjective factors during weight calculation. The multi-dimensional vector cosine method transforms the importance of customer requirement into a vector to express. This method combines multiple dimensions through the integration of vectors with objective mathematical method and avoids the interference of subjectivity on the calculation of final importance. Thus, the calculation results are more reliable.

## Case Study

Water in China is characterised by serious pollution and high level of water hardness. The household reverse osmosis membrane water purifier (hereinafter referred to as water purifier) is used for solving water problems in China. However, some problems in the process of using existing water purifier still trouble customers (Yu et al., 2018).

### Steps of the Case Study

Customer requirements for water purifiers are firstly obtained through market research, interviews and network reviews to determine the customer requirements for improving existing water purifiers. Then, questionnaires and expert interviews were designed to obtain importance data. Finally, the final importance is calculated. The case study involves following investigation and research steps.

Step 1: Obtain customer requirements.

The people investigated included water purifier distributors, salesmen, purchasers, users, and others. We conducted research of the distributors and salesmen of different brands of water purifiers. The requirements for improving existing water purifiers from distributors and salesmen were obtained through focus group interviews. According to the QQ and We Chat groups provided by water purifier distributors and salesmen, we directly contacted the users of water purifiers and obtained the original data provided by customers through issuing customer requirement tables, interviews, and observation methods. The second-hand data of customer requirements were obtained by sorting out buyers’ comments from online shopping platforms.

The requirements of buyers, users and distributors for improving existing water purifiers were obtained through the above-mentioned approaches. Thirty original customer requirements were obtained, as shown in Table 2.

**TABLE 2:** Original customer requirements of water purifier.

Original customer requirements	
Rapid water flow	With filter replacement reminder function
Waste as little water as possible	Low noise
Long replacement cycle of filter	Small volume
Remotely operated	Safe to use; no leakage of electricity
Real-time monitoring of water quality	Low maintenance cost
Space-saving	With waste water reuse function
Simple to operate	Fine workmanship
With alarming function	High filtration efficiency
Simple to wash	Secondary pollution can be avoided
Fast filtering	Easy maintenance
Self-cleaning function	Durable
Simple dismantling	Reducing power consumption
Low failure rate	Fast filtering speed
Intelligent operation	Filtered water has a good taste
Reasonable utilisation of kitchen space	With heating and refrigeration function

**FIGURE 9:**
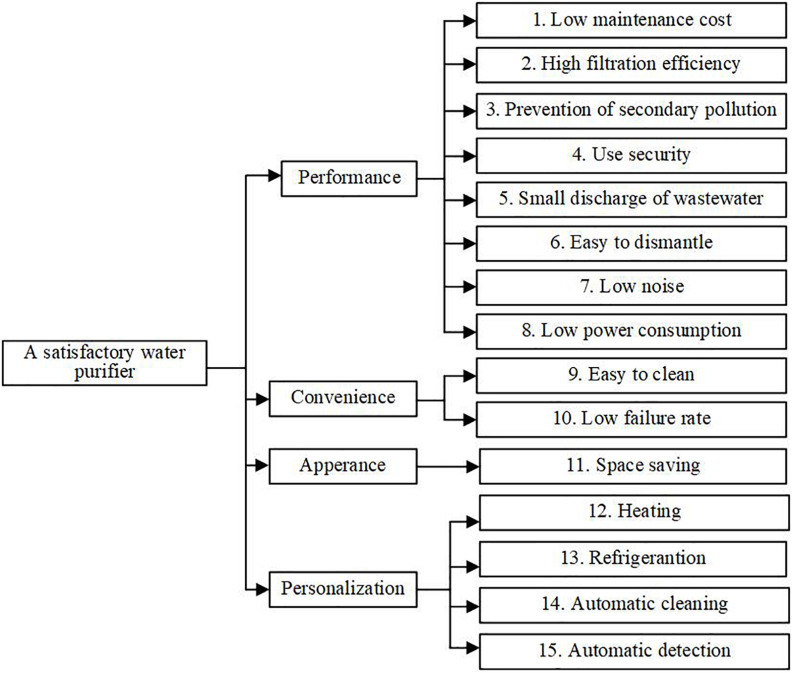
Tree chart of customer requirements for water purifier.

Step 2: Sort the original customer requirements, and form a tree chart (Figure 9).

Step 3: Design and distribute questionnaires to obtain the original importance and improvement of customer requirements from the customer’ s perspective.

(1) Design of questionnaires: Questionnaires are designed on the basis of the IGA, including positive, negative and current satisfaction questions. Forty-five questions were formed. The original importance and the improvement of each customer requirement are calculated according to the data provided by customers. Questionnaires were distributed mainly through the questionnaire website^1^ and 2D code. We also have direct access to some water purifier customers through the We Chat and QQ groups provided by friends who are dealers of water purifiers.

(2) Issuance and collection of formal questionnaires: The questionnaires are distributed using network address and 2D code generated through Wenjuan Xing. A total of 250 questionnaires were sent out, and 207 questionnaires were collected. Nine invalid questionnaires were excluded. The recovery rate of the questionnaires was 82.8%, and the effective rate was 79.2%.

(3) Data analysis and processing: After the questionnaires were collected, the data were analysed using IBM SPSS Statistics 19 software. Table 3 illustrates that the reliability coefficient is larger than 0.9, which shows that the questionnaires have high reliability. The questionnaires also have high structural validity, as summarised in Table 4.

**TABLE 3:** Reliability statistics of the questionnaires.

Cronbach’s Alpha	Cronbach’s Alpha based on Standardization	Item
0.947	0.951	45

**TABLE 4:** KMO and Bartlett test of the questionnaires.

Kaiser-Meyer-Olkin		0.909
Bartlett spherical test	Approximate chi-square	7741.832
	df	990
	Sig.	0.000

(4) Calculate improvement *W*_*IG*_ and original importance *W*_*AN*_ of the customer requirements from the perspective of customers according to Eqs. (1)–(6). The calculation results are shown in Table 5.

**TABLE 5:** Original importance and improvement of customer requirements.

Customer requirements	*AP*	*AN*	*ACS*	*IG*	*W*_*AN*_	*W*_*IG*_
1	4.57	4.1	3.4	1.17	0.0644	0.0844
2	4.59	4.28	3.56	1.03	0.0672	0.0743
3	4.67	4.21	3.38	1.29	0.0661	0.0931
4	4.69	4.49	3.83	0.86	0.0705	0.0620
5	3.6	4.42	3.4	0.2	0.0694	0.0144
6	4.61	4.45	3.78	0.83	0.0698	0.0599
7	4.71	4.4	3.81	0.9	0.0691	0.0649
8	4.71	4.43	3.77	0.94	0.0695	0.0678
9	4.71	4.39	3.69	1.02	0.0689	0.0736
10	4.59	4.33	3.64	0.95	0.0680	0.0685
11	4.69	4.35	3.86	0.83	0.0683	0.0599
12	4.47	3.91	3.59	0.88	0.0614	0.0635
13	4.37	3.85	3.56	0.81	0.0604	0.0584
14	4.6	3.99	3.5	1.1	0.0626	0.0794
15	4.61	4.11	3.56	1.05	0.0645	0.0758

Step 4: Calculate the basic importance of the customer requirements from the perspective of experts. Five experts on water purifiers were invited to evaluate the basic importance of customer requirements for water purifiers. Considering the differences in water quality in various regions, two sales managers, a senior manager, a technical researcher and an after-sales technician from different cities of China were chosen to form an expert group. Interval grey numbers are applied by the expert group to evaluate the basic importance of customer requirements, which is calculated using the improved interval grey number ranking method. The specific process is as follows:

(1) Determine the ideal interval grey numbers ⊗*G^∗^* 1 and divide them into different sets.

$$\begin{array}{l} ⊗G_{1}^{*}\\ =\left[\max\left({\underline{CR}}_{1},{\underline{CR}}_{2},\cdots ,{\underline{CR}}_{15}\right),\max\left({\bar{CR}}_{1},{\bar{CR}}_{2},\cdots ,{\bar{CR}}_{15}\right)\right]\\ =[0.71,0.\text{85}] \end{array}$$

All interval grey numbers representing the basic importance of customer requirements are compared with the ideal interval grey number. The interval grey number set ⊗*G_*A*_* is composed of the interval grey numbers satisfying the condition *CR_*i*_* > *G*^∗^ 1.

$$⊗G_{A}=\left\{⊗CR_{1},⊗CR_{2},⊗CR_{3},⊗CR_{4},⊗CR_{5},⊗CR_{6},⊗CR_{15}\right\}$$

The areas of the isosceles right triangles formed by the lines corresponding to the upper bounds of the interval grey numbers in the set ⊗*G_*A*_*, the lower bound of the ideal interval grey number and the line that forms a 45° angle with the vertical and horizontal axes are calculated according to Eq. (19). The calculation results are as follows:

$$\begin{array}{cc} S\left(⊗CR_{1}^{*},⊗G_{1}^{*}\right)=\frac{{\left(0.74-0.71\right)}^{2}}{2}=0.00045 & \\ S\left(⊗CR_{2}^{*},⊗G_{1}^{*}\right)=\frac{{\left(0.8-0.71\right)}^{2}}{2}=0.00405 & \\ S\left(⊗CR_{3}^{*},⊗G_{1}^{*}\right)=\frac{{\left(0.82-0.71\right)}^{2}}{2}=0.00605 & \\ S\left(⊗CR_{4}^{*},⊗G_{1}^{*}\right)=\frac{{\left(0.75-0.71\right)}^{2}}{2}=0.0008 & \\ S\left(⊗CR_{5}^{*},⊗G_{1}^{*}\right)=\frac{{\left(0.85-0.71\right)}^{2}}{2}=0.0098 & \\ S\left(⊗CR_{6}^{*},⊗G_{1}^{*}\right)=\frac{{\left(0.78-0.71\right)}^{2}}{2}=0.00245 & \\ S\left(⊗CR_{15}^{*},⊗G_{1}^{*}\right)=\frac{{\left(0.76-0.71\right)}^{2}}{2}=0.00125 & \end{array}$$

(2) Rank the interval grey numbers in set ⊗*G_B_*

Set ⊗*G_B_* is composed of interval grey numbers satisfying condition *CR_j_* < *G*^∗^_1_. The ideal interval grey number ⊗*G*^∗^_2_ in set ⊗*G_B_* is identified according to Eq. (22). The areas formed by the lines corresponding to the upper bounds of interval grey numbers, the lower bound of the ideal interval grey number and the 45° line are calculated. The calculation results of the areas of the corresponding isosceles right triangles are as follows:

$$\begin{array}{c} G_{2}^{*}=\left[\max\left({\underline{CR}}_{7},{\underline{CR}}_{8},\cdots ,{\underline{CR}}_{14}\right),\max\left({\bar{CR}}_{7},{\bar{CR}}_{8},\cdots ,{\bar{CR}}_{14}\right)\right]\\ =\left[0.3,0.51\right] \end{array}$$

$$\begin{array}{c} S\left(⊗CR_{7}^{*},⊗G_{2}^{*}\right)=\frac{{\left(0.40-0.3\right)}^{2}}{2}=0.005\\ S\left(⊗CR_{8}^{*},⊗G_{2}^{*}\right)=\frac{{\left(0.39-0.3\right)}^{2}}{2}=0.00405\\ S\left(⊗CR_{9}^{*},⊗G_{2}^{*}\right)=\frac{{\left(0.51-0.3\right)}^{2}}{2}=0.02205\\ S\left(⊗CR_{10}^{*},⊗G_{2}^{*}\right)=\frac{{\left(0.38-0.3\right)}^{2}}{2}=0.0032\\ S\left(⊗CR_{11}^{*},⊗G_{2}^{*}\right)=\frac{{\left(0.42-0.3\right)}^{2}}{2}=0.0072\\ S\left(⊗CR_{12}^{*},⊗G_{2}^{*}\right)=\frac{{\left(0.36-0.3\right)}^{2}}{2}=0.0018\\ S\left(⊗CR_{13}^{*},⊗G_{2}^{*}\right)=\frac{{\left(0.34-0.3\right)}^{2}}{2}=0.0008\\ S\left(⊗CR_{14}^{*},⊗G_{2}^{*}\right)=\frac{{\left(0.45-0.3\right)}^{2}}{2}=0.01125 \end{array}$$

(3) Standardise the areas corresponding to interval grey numbers in set ⊗*G_B_* using Eq. (23). Then, the basic importance of all customer requirements is calculated using Eq. (24). The result is shown in Table 6.

**TABLE 6:** Basic importance of customer requirements provided by experts.

Customer requirement	Expert 1	Expert 2	Expert 3	Expert 4	Expert 5	Evaluation result	Basic importance
1	[0.35,0.6]	[0.35,0.6]	[0.85,0.9]	[0.5,0.7]	[0.85,0.9]	[0.58,0.74]	0.01779
2	[0.85,1]	[0.4,0.5]	[0.8,0.95]	[0.25,0.55]	[0.75,1]	[0.61,0.8]	0.16008
3	[0.65,0.9]	[0.8,0.95]	[0.5,0.9]	[0.35,0.4]	[0.8,0.95]	[0.62,0.82]	0.23913
4	[0.55,0.85]	[0.4,0.5]	[0.6,0.85]	[0.15,0.2]	[0.35,0.6]	[0.41,0.75]	0.03162
5	[0.6,0.72]	[0.85,0.9]	[0.75,0.95]	[0.4,0.7]	[0.95,1]	[0.71,0.85]	0.38735
6	[0.7,0.8]	[0.6,0.85]	[0.7,0.8]	[0.25,0.4]	[0.45.0.5]	[0.54,0.78]	0.09684
7	[0.1,0.15]	[0.1,0.3]	[0.7,0.8]	[0.1,0.3]	[0.25,0.4]	[0.25,0.40]	0.00161
8	[0.1,0.2]	[0.15,0.2]	[0.6,0.7]	[0.25,0.35]	[0.3,0.5]	[0.28,0.39]	0.00130
9	[0.2,0.35]	[0.25,0.4]	[0.4,0.65]	[0.25,0.45]	[0.35,0.7]	[0.29,0.51]	0.00709
10	[0.2,0.25]	[0.1,0.2]	[0.45,0.5]	[0.15,0.35]	[0.45,0.6]	[0.27,0.38]	0.00103
11	[0.3,0.45]	[0.25,0.35]	[0.4.0.6]	[0.1,0.2]	[0.25,0.5]	[0.26,0.42]	0.00231
12	[0.25,0.3]	[0.05,0.15]	[0.2,0.5]	[0.25,0.35]	[0.15,0.5]	[0.18,0.36]	0.00058
13	[0.25,0.3]	[0.05,0.1]	[0.1,0.3]	[0.1,0.15]]	[0.25,0.35]	[0.15,0.34]	0.00026
14	[0.4,0.5]	[0.1,0.3]	[0.35,0.45]	[0.3,0.4]	[0.35,0.6]	[0.3,0.45]	0.00362
15	[0.35,0.45]	[0.5,0.6]	[0.5,0.6]	[0.25,0.45]	[0.55,0.7]	[0.43,0.76]	0.04941

Step 5: Calculate the final importance of customer requirements by using the multi-dimensional vector cosine method. The integration of the customer requirement vector in the ideal direction in the 3D space is calculated according to Eqs. (25)–(28); this is the final importance of customer requirements. The calculation results are listed in Table 7.

**TABLE 7:** Final importance of customer requirements for water purifier.

No. of Customer requirement	Final Importance	Ranking	No. of Customer requirement	Final importance	Ranking
1.	0.10763	6	9.	0.10105	8
2.	0.18884	3	10.	0.09650	11
3.	0.26499	2	11.	0.09085	13
4.	0.09905	9	12.	0.08831	14
5.	0.39378	1	13.	0.08399	15
6.	0.13355	4	14.	0.10115	7
7.	0.09479	12	15.	0.11110	5
8.	0.09708	10			

### Analysis of the Data

In Figure 8, the X, Y, and Z axes represent the customer requirement’s original importance, basic importance and improvement, respectively, which are taken as the three dimensions in the 3D space. According to the case study of the above-mentioned five steps, the importance of customer requirement obtained from different dimensions is different, which is ignored by the existing literature. The original importance of customer requirement is different from the improvement, which is the proof of the view proposed in this research. Meeting the customer requirement with high original importance may not be able to improve customer satisfaction to the greatest extent. Therefore, we cannot carry out incremental product innovation simply according to the original importance of customer requirement.

We obtain three dimensions of customer requirement importance data from experts and customers. According to Table 5, from the perspective of the original importance of customer requirement, the most important customer requirement is the safety in use. The safety includes not only the personal safety of customers when using the water purifier but also the water purifier that does not leak, does not soak the kitchen floor, does not bring hidden dangers to the family and so on. From the perspective of whether it is important or not, customers pay more attention to the safety of the water purifier. From the perspective of the basic importance of customer requirement, the most important customer requirement is the small discharge of wastewater. The importance of customer requirement disclosed by industry experts is different from that provided by customers, precisely because customers are more likely to consider problems from their own short-term needs. Customers’ feelings and needs easily change and will frequently change with the progress of technology. Enterprises develop products that customers need through a long R&D cycle to meet the needs of customers. When customers easily change their original requirements and produce new ones, the market of innovative products is cold, and the innovation fails.

Therefore, the calculation of customer requirements is inseparable from industry experts who tend to measure the importance of customer requirements from the long-term sustainable development of products.

The 3D vector cosine method is used to objectively calculate through the data itself to integrate the three dimensions of customer requirement importance and calculate the final importance of customer requirement. According to the final importance data, the top three customers’ demand for water purifiers are small discharge of wastewater, prevention of secondary pollution and high filtration efficiency. This notion provides a basis for water purifier manufacturers to realise the incremental innovation of water purifiers.

### Comparisons of the Methods

#### Comparison of the Improved Interval Grey Number Ranking Method With Other Methods

The three methods for ranking interval grey numbers are as follows: set theory-based, probability function-based and ideal interval grey number-based methods. The set theory-based method determines the rank of interval grey numbers by comparing the upper bounds, lower bounds, intermediate values and widths. For example, the following formulas are used to solve the maximisation problem (Sengupta and Pal, 2009):

⊗Ga<⊗Gb,ifGa¯<Gb¯⊗Ga≤⊗Gb,ifGa¯≤Gb¯,Ga¯≤Gb¯⊗Ga≤⊗Gb,ifm(⊗Ga)≤m(⊗Gb),w(⊗Ga)≤w(⊗Gb)⊗Ga≤⊗LmGb,ifm(⊗Ga)≤m(⊗Gb),Ga¯≤Gb¯

where *m*, *w*, and *Lm* represent the intermediate value, the width and the lower bound of the interval grey number, respectively. This method immediately provides the comparison results; however, it cannot rank the interval grey numbers with intersecting relationships.

Taking [3,7] and [2,9] as examples, the two interval grey numbers intersect with each other. The comparison result of the upper bounds is opposite to that of the lower bounds. Meanwhile, the comparison result of the intermediate value is also opposite to that of the lower bounds. Therefore, the ranking result of the two interval grey numbers cannot be obtained using the above formulas with a set theory-based method.

Nakahara et al. (1992) proposed the concept of unequal probability to rank two different interval grey numbers. On this basis, Wang et al. (2014) proposed the probability function to rank two interval grey numbers. The probability function is set to gain the possibility of one interval grey number larger than the other one. The possibility degree function is artificially determined. Therefore, the comparison result is subjective to a certain extent.

Liu and Cheng (2016) used the ideal interval grey number as a measure standard to compare two interval grey numbers. The larger upper and lower bounds of the two interval grey numbers are selected for the maximisation problem to form an ideal interval grey number. The smaller upper and lower bounds of the two interval grey numbers are selected for the minimisation problem to form an ideal interval grey number. A triangle is formed by the lines corresponding to the upper bound of the interval grey number, the lower bound of the ideal interval grey number and the line whose angles with the vertical and horizontal axes are 45°. The two interval grey numbers are compared on the basis of the areas of the corresponding triangles. The interval grey number with a larger area is prior.

A common point of the three methods mentioned above is that they are limited to the comparison between two interval grey numbers in a single time. Therefore, comparing multiple interval grey numbers is complicated.

In this research, an improved ranking method for interval grey numbers is proposed. With the proposed method, the ideal interval grey number is firstly selected amongst all interval grey numbers. Then, all interval grey numbers are divided into two categories on the basis of their relationships with the ideal interval grey number. Different ranking steps are proposed for each category of interval grey numbers. The method can simultaneously compare multiple interval grey numbers at a time. The improved method effectively reduces the comparison times of the interval grey numbers and simplifies the calculation process. The weights of all interval grey numbers can also be calculated with the method. The comparison of all the methods is shown in Table 8.

**TABLE 8:** Comparison of the ranking methods for interval grey numbers.

Method	Proposer	Number of interval grey numbers for each comparison	Comparison times of the basic importance of customer requirement in the case study
Based on set theory	Sengupta and Pal (2009)	Two	105
Based on probability function	Nakahara et al. (1992); Wang (2012)	Two	105
Based on ideal interval grey number	Liu and Cheng (2016)	Two	105
Proposed method	This literature	More than two (one group)	2

Experts use interval grey number to assign the importance of customer requirement to solve the uncertainty during customer requirement acquisition. The data in the case study are used in the verification stage to illustrate the advantages and convenience of the proposed method. The data in Table 6 illustrate that 15 customer requirement importance intervals provided by experts are sorted by this method. The following results are obtained: ⊗*G*_5_ > ⊗*G*_3_ > ⊗*G*_2_ > ⊗*G*_6_ > ⊗*G*_15_ > ⊗*G*_4_ > ⊗*G*_1_ > ⊗*G*_9_ > ⊗*G*_14_ > ⊗*G*_11_ > ⊗*G*_7_ > ⊗*G*_8_ > ⊗*G*_10_ > ⊗*G*_12_ > ⊗*G*_13_. The method proposed by Liu and Cheng (2016) is used to verify the results. The verification process is shown in Table 9. The results are consistent with those calculated by the method proposed by Liu and Cheng.

**TABLE 9:** Verification of the calculating results.

Interval grey number	Idea Interval grey number	S(⊗*G, ⊗G^∗^*)	Ranking result
⊗*G*_5_ vs⊗*G*_3_	[0.71,0.85]	S(⊗*G*_5_, ⊗*G*1^∗^):0.0098 *S*(⊗*G*_3_, ⊗*G*^∗^):0.00605	⊗*G*_5_ > ⊗*G*_3_
⊗*G*_3_ vs⊗*G*_2_	[0.62,0.82]	S(⊗*G_3_*,⊗*G^∗^*):0.02000 S(⊗*G_2_*,⊗*G^∗^*):0.01620	⊗*G*_3_ > ⊗*G*_2_
⊗*G*_2_vs⊗*G*_6_	[0.61,0.8]	S(⊗*G_2_*,⊗*G^∗^*):0.01805 S(⊗*G_6_*,⊗*G^∗^*):0.01445	⊗*G*_2_ > ⊗*G*_6_
⊗*G*_6_vs⊗*G*_15_	[0.54,0.78]	S(⊗*G_6_*,⊗*G^∗^*):0.02880 S(⊗*G_15_*,⊗*G^∗^*):0.02420	⊗*G*_6_ > ⊗*G*_15_
⊗*G*_15_ vs⊗*G*_4_	[0.43,0.76]	S(⊗*G*_15_,⊗*G^∗^*):0.05445 S(⊗*G_4_*,⊗*G^∗^*):0.05120	⊗*G*_15_ > ⊗*G*_4_
⊗*G*_4_vs⊗*G*_1_	[0.58,0.75]	S(⊗*G_4_*,⊗*G^∗^*):0.01445 S(⊗*G_1_*,⊗*G^∗^*):0.01280	⊗*G*_4_ > ⊗*G*_1_
⊗*G*_1_vs⊗*G*_9_	[0.58,0.74]	S(⊗*G_1_*,⊗*G^∗^*):0.01280 S(⊗*G_9_*,⊗*G^∗^*):0.00245	⊗*G*_1_ > ⊗*G*_9_
⊗*G*_9_ vs⊗*G*_14_	[0.3,0.51]	S(⊗*G_9_*,⊗*G^∗^*):0.02205 S(⊗*G_14_*,⊗*G^∗^*):0.01125	⊗*G*_9_ > ⊗*G*_14_
⊗*G*_14_vs⊗*G*_11_	[0.3,0.45]	S(⊗*G_14_*,⊗*G^∗^*):0.01125 S(⊗*G_11_*,⊗*G^∗^*):0.00720	⊗*G*_14_ > ⊗*G*_11_
⊗*G*_11_vs⊗*G*_7_	[0.26,0.42]	S(⊗*G_11_*,⊗*G^∗^*):0.01280 S(⊗*G_7_*,⊗*G^∗^*):0.00980	⊗*G*_11_ > ⊗*G*_7_
⊗*G*_7_vs⊗*G*_8_	[0.28,0.40]	S(⊗*G_7_*,⊗*G^∗^*):0.00720 S(⊗*G_8_*,⊗*G^∗^*):0.00605	⊗*G*_7_ > ⊗*G*_8_
⊗*G*_8_vs⊗*G*_10_	[0.28,0.39]	S(⊗*G_8_*,⊗*G^∗^*):0.00605 S(⊗*G_10_*,⊗*G^∗^*):0.00500	⊗*G*_8_ > ⊗*G*_10_
⊗*G*_10_vs⊗*G*_12_	[0.27,0.38]	S(⊗*G_10_*,⊗*G^∗^*):0.00605 S(⊗*G_12_*,⊗*G^∗^*):0.00405	⊗*G*_10_ > ⊗*G*_12_

However, according to Eq. (33), a comparison must be conducted for 105 times to determine the ranking results of grey numbers of 15 customer requirement intervals by using existing methods because only two interval grey numbers are compared at a time. The method proposed in this study only uses two steps and compares twice, and the ranking results of 15 interval grey numbers are obtained.

(33) $${\text{C}}_{15}^{2}=\frac{A_{15}^{2}}{A_{2}}=\frac{15\times 14}{2}=105$$

#### Comparison Between the Integrated Method for Calculating the Final Customer Requirement Importance and Other Methods

Table 10 shows the comparison between the integrated method for calculating the final customer requirement importance and other methods. This integrated method proposed in this research has the following advantages compared with other methods: Firstly, the concept of customer requirement improvement is proposed for the first time on the basis of the gap between customer satisfaction with current and ideal products according to the characteristics of product incremental innovation. This concept takes the improvement for customer satisfaction to meet a certain customer requirement as an important index to measure the importance of customer requirement. Thus, the effect of incremental product innovation can improve customer satisfaction to a greater extent. Secondly, we integrate the importance of customer requirement from three dimensions, including basic importance, original importance, and improvement. The multi-dimensional vector cosine method is proposed to calculate the final importance to objectively assign the weight of dimension data. This mechanism ensures the objectivity and scientificity of the data in the calculation process. However, the existing methods subjectively regard the importance of different dimensions of customer requirements as equal. The calculation of final customer requirement importance using the methods in existing literature only considers the numerical value of every dimension, but the difference of the weights is ignored.

**TABLE 10:** Comparison between the proposed method and other methods.

Literature	Dimensions				Method for integrating different dimensions
	Original importance	Improvement		Basic importance	
		Current satisfaction	Ideal product’ satisfaction		
Haber et al. (2018)	√				Subjective
Zheng et al. (2019)	√				Subjective
Li et al. (2018)	√			√	Subjective
Sun et al. (2020)	√			√	Subjective
Gupta and Shri (2018)	√	√			Subjective
The proposed method	√	√	√	√	Objective

The multi-dimensional vector cosine method integrates the three dimensions of customer requirements objectively and considers the numerical value and weight of each dimension. This method is more reliable than existing methods.

## Discussion

This research studied the integrated calculation process of the importance of customer requirements for incremental product innovation from the following aspects:

Firstly, customer requirements for incremental product innovation refer to the requirements for improving existing products. To more effectively distinguish the importance of customer requirements, importance is measured by customer perceived satisfaction and dissatisfaction, which is more in line with the law of human perception. Accordingly, the direction for improving existing products from the true feelings of customers is identified. The IGA method is used to set negative questions for measuring the dissatisfaction perceived by customers when existing products do not meet customer requirements. The more unsatisfied customers feel, the more important is the requirement. Thus, the original importance of customer requirement depends on the dissatisfaction customers perceived.

Experts are invited to evaluate the basic importance of customer requirements from the perspective of product innovation because they have a deeper and systematic understanding of industry development and the directions of product innovation. This initiative is proposed to obtain customer requirement importance data from the perspectives of customers and experts. On the one hand, customers’ requirements are considered from the perspective of the market. On the other hand, the prospects of product innovation are considered from the perspective of product, which is conducive to the long-term development of products.

Secondly, the gaps between existing products and ideal products are analysed to provide the direction of incremental product innovation. The concept of improvement is suggested for the first time as one of the indicators to measure the final importance of customer requirements. If a customer requirement is satisfied by a newly innovated product, then the higher customer satisfaction is improved by meeting this requirement, the higher the improvement of this customer requirement is. This situation can effectively suggest a direction of product innovation and significantly improve customer satisfaction by integrating the improvement provided by customers into the calculation process of the final importance of customer requirements.

Thirdly, the evaluation data provided by experts are often inadequate because of the limitation of experts’ number, which results in the uncertainty of expert evaluation data. To solve this problem, interval grey numbers are used by experts for evaluation. The interval grey number is a form of expression in grey theory, which effectively solves the uncertain problem of inadequate data and less information. In this study, an improved interval grey number ranking method is proposed, which classifies multiple interval grey numbers into different categories on the basis of their relationships with the ideal interval grey number. This method can simultaneously compare multiple interval grey numbers of each category and effectively reduce the comparison times of interval grey numbers. The ranking of interval grey numbers is faster and more accurate.

Fourthly, the original importance, the improvement obtained from the customers’ perspective and the basic importance acquired from the experts’ perspective are comprehensively integrated to calculate the final importance of customer requirements. Accordingly, the calculation results reflect the requirements of the customers and significantly improve customer satisfaction with incremental product innovation. A multi-dimensional vector cosine method is proposed. This method considers customer requirement as a vector in a 3D space. The lengths and angles deviating from the ideal direction of customer requirement vectors are considered to calculate the projection of the customer requirement vector in the ideal direction, which is the final importance of customer requirements. This method objectively realises the integration calculation of customer requirements’ importance and further improves the reliability of the customer-requirement driven process in incremental product innovation.

## Conclusion

### Managerial Implications

For enterprises, incremental product innovation is the source of development and progress, and enterprises without incremental product innovation will be replaced by other advanced ones. One of the key factors that determine the success of product innovation is whether the innovation meets the most important requirements of customers. Product innovation based on meeting the needs of customers is the key to win market share and improve competitiveness. Therefore, the driving force of customer requirements must be fully reflected to accomplish incremental product innovation. The first step of implementing a customer requirement-driven process is to identify customer requirements, which means customer requirements for improvement of existing products. Incremental product innovation is the innovation of existing products. The problems of existing products can be identified according to the improvement requirements of customers. Under the restriction of resources and ability, enterprises should find the product problems that customers pay most attention to. The importance of customer requirements must be accurately calculated to determine the problems to be solved in incremental product innovation for effectively distinguishing the seriousness of the problems of existing products.

In contrast with previous studies, this work achieves the effective integration of customer requirement importance from three dimensions. The importance data provided by customers and experts are integrated to meet customers’ requirements and the development prospects of the industry. The concept of customer requirement improvement is proposed for the first time to effectively identify the key problems existing in existing products. The improvement of customer requirement is calculated and integrated into the calculation process of customer requirement importance by measuring the gap between the existing products and the ideal products, which makes the incremental product innovation driven by customer requirements effective to a greater extent. Hence, the effect of incremental product innovation can improve customer satisfaction. This study can help enterprises in fully understanding the market requirement and the development trend of products and provide the basis for enterprises to realise incremental product innovation. Therefore, enterprises can continuously realise product incremental innovation and occupy an advantage in the market competition.

### Limitations

Despite the positive contributions, this study certainly presents some limitations. Firstly, the integrated method proposed in this study theoretically divides the calculation of customer requirements into three dimensions. The study verified that the importance of customer requirements obtained from different dimensions is different. The data in the case study are only used to verify the improved interval grey number ranking method and the integrated method are not verified because the existing research does not fully consider the three dimensions. Secondly, this study only proposes three dimensions of customer requirement importance calculation, which is a supplement to the existing literature. The other dimensions of customer requirements remain to be further studied and verified in the future. Thirdly, the research method proposed in this study only provides the basis and direction for the realisation of incremental product innovation by calculating the importance of customer requirements. This phase is only the early stage of product incremental innovation, and the follow-up product design needs further research.

This research demonstrated the calculation process of the importance of customer requirements from the perspectives of experts and customers. Three dimensions, including the original importance, improvement and basic importance, were considered to calculate the final importance of customer requirements. This research provided a basis for the driven process of customer requirements in incremental product innovation. Future research should aim to study the role of the importance of customer requirements in the following detailed design of the incremental product innovation process.

## Data Availability Statement

The raw data supporting the conclusions of this article will be made available by the authors, without undue reservation.

## Author Contributions

LG: conceptualization, methodology, and writing – original draft. XS: formal analysis. MC: funding acquisition. LZ: investigation. XS, LG, and JX: supervision. LZ and JX: validation. LG, XS, LZ, MC, and JX: writing – review & editing. All authors contributed to the article and approved the submitted version.

## Conflict of Interest

The authors declare that the research was conducted in the absence of any commercial or financial relationships that could be construed as a potential conflict of interest.

## Footnotes

1 https://www.wjx.cn/jq/19202974.aspx
